# Association of carotid intima-media thickness with the risk of sudden sensorineural hearing loss

**DOI:** 10.7717/peerj.9276

**Published:** 2020-06-03

**Authors:** Chun-Hsien Ho, Teng-Yeow Tan, Chung-Feng Hwang, Wei-Che Lin, Ching-Nung Wu, Chao-Hui Yang

**Affiliations:** 1 Department of Otolaryngology, Kaohsiung Chang Gung Memorial Hospital and Chang Gung University College of Medicine, Kaohsiung, Taiwan; 2 Department of Neurology, Kaohsiung Chang Gung Memorial Hospital and Chang Gung University College of Medicine, Kaohsiung, Taiwan; 3 Department of Diagnostic Radiology, Kaohsiung Chang Gung Memorial Hospital and Chang Gung University College of Medicine, Kaohsiung, Taiwan

**Keywords:** Sudden sensorineural hearing loss, Carotid intima-media thickness, Atherosclerosis

## Abstract

Cardiovascular factors are associated with the pathophysiological features and risk of sudden sensorineural hearing loss (SSNHL). However, little is known about the link between carotid intima-media thickness (IMT), SSNHL risk, and their respective treatment outcomes. In this study, we retrospectively reviewed 47 SSNHL cases and 33 control subjects from a single medical center and compared their demographic data and clinical characteristics, including their carotid IMT and audiological data. Of the 80 enrolled subjects, the proportion of those with high carotid IMT was greater in the SSNHL group (53.2%) than in the control group (21.2%), with an odds ratio (OR) of 4.22 (95% confidence interval (CI) [1.53–11.61], *P* = 0.004). Notably, high carotid IMT was more common in female SSNHL patients than females in the control group (54.2% vs. 12.5%; OR, 8.27 (95% CI [1.53–44.62]), *P* = 0.008), particularly in female patients ≥50 years of age (75% vs. 25%; OR, 9.0 (95% CI [1.27–63.9]), *P* = 0.032). The multivariate regression analyses showed the association between high carotid IMT and SSNHL with an adjusted OR of 4.655 (95% CI [1.348–16.076], *P* = 0.015), particularly in female SSNHL patients (adjusted OR, 9.818 (95% CI [1.064–90.587], *P* = 0.044). The carotid IMT was not associated with the treatment outcomes of SSNHL. Our results indicate that early-stage atherosclerosis may be associated with SSNHL, particularly in female patients more than 50 years old.

## Introduction

Sudden sensorineural hearing loss (SSNHL) is defined as a hearing loss of over 30 dB in three sequential frequencies developing within 3 days. The annual incidence is about 5–20 out of 100,000 people. SSNHL was previously thought to be mostly idiopathic, as the cause was often unidentified. Several etiological hypotheses, including vascular insults, membrane breaks and viral cochleitis have been proposed (Merchant, Adams & Nadol, 2005). Of the hypothesized etiologies, vascular ischemia or occlusion is one of the most popular. The internal auditory artery supplies blood to the inner ear, which lacks collateral circulation (Merchant, Adams & Nadol, 2005). Previous studies have reported several cardiovascular risk factors for SSNHL. For example, the rates of diabetes mellitus (DM) and hypercholesteremia were higher in SSNHL patients than those in the control group (Aimoni et al., 2010). Metabolic syndrome increased the risk of SSNHL in Taiwan (Chien et al., 2015) and Korea (Jung et al., 2018). Studies also showed that the risk of stroke was higher in SSNHL patients (Kim, Hong & Kim, 2018a; Lin, Chao & Lee, 2008) and vise versa (Kuo et al., 2016).

Common carotid artery (CCA) intima-media thickness (IMT), measured via Doppler ultrasonography, has frequently been used as a surrogate marker of subclinical atherosclerosis in many epidemiological and interventional studies of cardiovascular and cerebrovascular diseases (Bots et al., 1997). However, the association between IMT and SSNHL has not been fully explored. Higher carotid IMT values were found in SSNHL patients (Mutlu et al., 2018; Rajati et al., 2016), suggesting an association between subclinical atherosclerosis and SSNHL (Rajati et al., 2016). However, IMT is affected by several factors. We previously reported an association between gender and carotid IMT (Tan et al., 2009). It remains unknown whether subclinical atherosclerosis of the carotid artery is associated with SSNHL in both males and females and whether IMT values are higher for both male and female SSNHL patients. Since the risk of stroke is one of the greatest concerns for SSNHL patients (Lin, Chao & Lee, 2008), it is vital to to explore the associations between carotid IMT and SSNHL using subgroup analyses.

Furthermore, the associations between cardiovascular risk factors and SSNHL treatment outcomes remain unclear. Hypertension (HTN) (Edizer et al., 2015), DM (Weng et al., 2005), and thrombophilia risk factors (Passamonti et al., 2015) have been linked to poor SSNHL prognosis. However, one study found that cardiovascular risk factors did not influence SSNHL hearing recovery (Ciorba et al., 2015). Since carotid IMT has been reported as a stroke severity marker (Heliopoulos et al., 2009), the association between carotid IMT and SSNHL treatment outcomes should be further examined.

We aimed to explore the potential association between carotid IMT and SSNHL and to analyze whether the results differed by sex. Additionally, this is the first investigation of the associations between carotid IMT and SSNHL treatment outcomes.

## Materials and Methods

### Subjects

We retrospectively enrolled idiopathic SSNHL patients admitted to Kaohsiung Chang Gung Memorial Hospital, Taiwan between September 2011 and December 2015. We also age- and sex-matched control subjects without a history of SSNHL from a neurological health examination center. All subjects underwent carotid Doppler ultrasonography. SSNHL was defined as a SSNHL ≥ 30 dB over at least three contiguous frequencies developed within 72 h. We excluded patients with SSNHL whose origins were known, such as retrocochlear lesions (detected by acoustic brainstem response and magnetic resonance imaging) and infectious or autoimmune disease (revealed by laboratory data). Bilateral SSNHL cases were also excluded from our study. All patients underwent SSNHL treatment, including the administration of intravenous corticosteroids (5 mg of dexamethasone every 6 h for 2 days, followed by 5 mg every 8 h for 2 days, and then 5 mg every 12 h for 1 day) and a plasma expander (500 mL of Dextran 40 (a low-molecular weight dextran) each day for 5 days). We recorded their clinical data, including age, sex, smoking status, DM or HTN history, total cholesterol, low-density lipoprotein (LDL) cholesterol and high-density lipoprotein (HDL) cholesterol levels. The Institutional Review Board of Chang Gung Memorial Hospital reviewed and approved the study protocol (reference number: 201801815B0).

### IMT measurement

The protocol for measuring carotid IMT has been previously described (Tan et al., 2009). A B-mode ultrasound system (Philips HDI 5000 System; ATL-Philips, Bothell, WA, USA) equipped with a 4–10 MHz linear array transducer was used to obtain longitudinal images of the common carotid artery. An ultrasound technologist blinded to all clinical information performed the examinations. Both the left and right CCA were routinely scanned along a 1-cm segment immediately proximal to the dilatation of the bifurcation plane. The images were transferred to a workstation and the IMT, defined as the distance between the interfaces of the lumen intima and media adventitia, was automatically measured using Q-LAB software (ATL-Philips, Amsterdam, Netherlands). All measurements were performed in a single-blinded fashion. The average carotid IMT was calculated as the mean of the left and right CCA IMT values. When evaluating the association between IMT and SSNHL, an average carotid IMT <0.65 mm was defined as low and a value ≥0.65 mm was considered high (Tan et al., 2012).

### Acoustic evaluation

Audiometry was performed using a GSI 61 audiometer (Grason-Stadler, Eden Prairie, MN, USA) and TDH-50p earphones (Telephonics, Farmingdale, NY, USA) in a soundproof booth. The pure-tone average (PTA) was the mean of the thresholds at 500, 1,000, 2,000 and 4,000 Hz. SSNHL was defined as hearing loss without an air-bone gap at each tested frequency. The word recognition score (WRS) was also evaluated by calculating the percentage of correctly identified words from a list of phonetically balanced, monosyllabic words. Pre- and post-treatment audiometry refer to hearing tests performed on the day of admission and about 3 months after treatment commenced, respectively. Disease severity was evaluated using the affected ear’s initial PTA and WRS values in addition to associated vertigo. The outcomes of SSNHL treatments were assessed by calculating the hearing gain and WRS change, which were defined as the difference in hearing thresholds and WRSs, respectively, between pre- and post-treatment audiometry. Additionally, Siegel’s criteria (Siegel, 1975) were used to evaluate the extent of hearing recovery.

### Statistical analysis

We used SPSS software for Windows (ver. 18.0; SPSS, Inc., Chicago, IL, USA) for statistical analyses. A *P-*value < 0.05 was considered to reflect statistical significance. The data means (with standard deviations, SDs),medians (with interquartile ranges, IQRs), and percentages were presented. The Kolmogorov–Smirnov test was used to assess whether parameters were normally distributed. Continuous variables, including age, cholesterol (total, LDL and HDL) levels, and average carotid IMT were compared between the SSNHL and control groups using an independent *t*-test for parametrically distributed data (age, total cholesterol and LDL cholesterol) or the Mann–Whitney *U*-test for non-parametrically distributed data (HDL cholesterol and average IMT). Categorical variables, including sex, smoking status, DM or HTN history and high/low IMT, were compared using Pearson’s chi-squared test or Fischer’s exact test (the latter when any expected value was less than 5). The categorical variable effect sizes were expressed as odds ratios (ORs) with 95% confidence intervals (CIs), which were calculated in the cross tables of a chi-square test for univariate analyses. The adjusted ORs in multiple logistic regression analyses were calculated using stepwise selection for age, HTN, LDL cholesterol and high average IMT.

## Results

### Carotid IMT in SSNHL and control subjects

We enrolled a total of 80 subjects (40 males (50%) and 40 females (50%); mean (SD) age, 51.8 (12.6) years (range, 20–80 years)), including 47 SSNHL patients and 33 participants in the control group. Their clinical characteristics are shown in Table 1. There were no significant between-group differences in sex or age. There were no significant differences in cardiovascular factors including smoking status, DM and HTN history, or lipid profiles (total, LDL and HDL cholesterol levels). However, SSNHL patients exhibited a significantly higher average carotid IMT (median, 0.65 mm; IQR, 0.55–0.83 mm) than in the control group (median, 0.58 mm; IQR, 0.55–0.64 mm) (*P* = 0.022). High carotid IMT (IMT ≥ 0.65 mm) was significantly more common in the SSNHL group (53.2%, 25 of 47) than the control group (21.2%, 7 of 33) (OR, 4.22; 95% CI [1.53–11.61]) (*P* = 0.004). After adjusting for age, HTN and LDL cholesterol, the multivariate analysis still showed an association between high carotid IMT and SSNHL, with an adjusted OR of 4.655 (95% CI [1.348–16.076], *P* = 0.015).

**Table 1:** Characteristics of SSNHL and control subjects. SSNHL patients had significant higher average IMT value and more percentage of high average IMT (≥0.65 mm) than those in the control group. *P* values <0.05 are considered statistically significant and are bolded.

	SSNHL (*n* = 47)	Control (*n* = 33)	*P* Value	OR (95% CI)^a^
Sex, *n* (%)				
Male	23 (48.9%)	17 (51.5%)	0.820	0.90 [0.37–2.20]
Female	24 (51.1%)	16 (48.9%)		1 [Reference]
Age, mean (SD), year	53.5 (13.2)	49.4 (11.5)	0.146	
Smoking, *n* (%)				
Yes	7 (14.9%)	3 (9.1%)	0.512	1.75 [0.42–7.33]
No	40 (85.1%)	30 (90.9%)		1 [Reference]
DM, *n* (%)				
Yes	9 (19.1%)	4 (12.1%)	0.402	1.72 [0.48–6.13]
No	38 (80.9%)	29 (87.9%)		1 [Reference]
HTN, *n* (%)				
Yes	17 (36.2%)	7 (21.2%)	0.151	2.10 [0.76–5.87]
No	30 (63.8%)	26 (78.8%)		1 [Reference]
Total cholesterol, mean (SD), mg/dl	196.7 (36.3)	193 (32.9)	0.688	
LDL cholesterol, mean (SD), mg/dl	121.5 (33.1)	108 (29.6)	0.117	
HDL cholesterol, median (IQR), mg/dl	56 (49.25–68)	56 (46.5–71.5)	0.994	
Average IMT, median (IQR), mm	0.65 (0.55–0.83)	0.58 (0.55–0.64)	**0.022**	
Average IMT, *n* (%)				
High (≥0.65 mm)	25 (53.2%)	7 (21.2%)	**0.004**	4.22 [1.53–11.61]
Low (<0.65 mm)	22 (46.8%)	26 (78.8%)		1 [Reference]

**Notes:**

SSNHL, sudden sensorineural hearing loss; DM, diabetes mellitus; HTN, hypertension; LDL, low-density lipoprotein; HDL, high-density lipoprotein; IMT, intima-media thickness; OR, Odds ratio; CI, confident interval.

a Crude OR in univariate analyses.

### Carotid IMT in SSNHL and control subjects stratified by sex

We then compared the cardiovascular risk factors and carotid IMT values between SSNHL and control subjects stratified by sex (Table 2). There was no significant difference in the number of subjects with a history of DM or HTN or in the total, LDL and HDL cholesterol levels between the SSNHL and control groups in females or males. However, a significantly higher average carotid IMT was evident in female SSNHL patients (median, 0.67 mm; IQR, 0.55–0.89 mm) compared to those in the control group (median, 0.56 mm; IQR, 0.55–0.63 mm) (*P* = 0.044). Likewise, significantly more female SSNHL patients had a higher IMT (54.2%, 13 of 24) compared to those in the control group (12.5%, 2 of 16; OR 8.27 (95% CI [1.53–44.62])) (*P* = 0.008). A high IMT was also linked to SSNHL in females after further adjusting for age, HTN and LDL cholesterol level in the multivariate analysis, with an adjusted OR of 9.818 (95% CI [1.064–90.587], *P* = 0.044). In contrast, the median IMT value for male SSNHL patients and the percentage of male SSNHL patients with high IMT values were not significantly different than those in the control group.

**Table 2:** Characteristics of SSNHL and control subjects stratified by sex. Female SSNHL patients had significant higher average IMT value and more percentage of high average IMT (≥0.65 mm) than those in the female control group. *P* values <0.05 are considered statistically significant and are bolded.

	SSNHL	Control	*P* Value	OR (95% CI)^a^
Age, mean (SD), year				
Female	53.6 (14.7)	47.6 (12.7)	0.167	
Male	53.9 (11.7)	51 (10.3)	0.561	
Smoking, *n* (%)				
Male				
Yes	7 (30.4%)	3 (17.6%)	0.471	2.04 [0.44–9.44]
No	16 (69.6%)	14 (82.4%)		1 [Reference]
DM, *n* (%)				
Female				
Yes	4 (16.7%)	2 (12.5%)	1.000	1.40 [0.22–8.72]
No	20 (83.3%)	14 (87.5%)		1 [Reference]
Male				
Yes	5 (21.7%)	2 (11.8%)	0.677	2.08 [0.35–12.3]
No	18 (78.3%)	15 (88.2%)		1 [Reference]
HTN, *n* (%)				
Female				
Yes	6 (25%)	3 (18.8%)	0.717	1.44 (0.3 to 6.87)
No	18 (75%)	13 (81.2%)		1 [Reference]
Male				
Yes	11 (47.8%)	4 (23.5%)	0.117	2.98 (0.74 to 11.93)
No	12 (52.2%)	13 (76.5%)		1 [Reference]
Total cholesterol, mean (SD), mg/dl				
Female	197.3 (34.7)	194.3 (38.7)	0.827	
Male	196 (39)	192.1 (29.3)	0.754	
LDL cholesterol, mean (SD), mg/dl				
Female	117.2 (36.5)	98 (28.1)	0.149	
Male	126.2 (28.9)	117.1 (29.2)	0.406	
HDL cholesterol, median (IQR), mg/dl				
Female	57 (53–69)	69 (47.5–94.25)	0.378	
Male	53 (45.5–66)	51 (46–57)	0.812	
Average IMT, median (IQR), mm				
Female	0.67 (0.55–0.89)	0.56 (0.55–0.63)	**0.044**	
Male	0.65 (0.55–0.83)	0.60 (0.54–0.70)	0.194	
Average IMT, *n* (%)				
Female				
High (≥0.65 mm)	13 (54.2%)	2 (12.5%)	**0.008**	8.27 [1.53–44.6])
Low (<0.65 mm)	11 (45.8%)	14 (87.5%)		1 [Reference]
Male				
High (≥0.65 mm)	12 (52.2%)	5 (29.4%)	**0.15**	2.62 [0.70–9.86]
Low (<0.65 mm)	11 (47.8%)	12 (70.6%)		1 [Reference]

**Notes:**

Smoking was not listed in females because all women were nonsmokers.

SSNHL, sudden sensorineural hearing loss; DM, diabetes mellitus; HTN, hypertension; LDL, low-density lipoprotein; HDL, high-density lipoprotein; IMT, intima-media thickness; OR, Odds ratio; CI, confident interval.

a stratified by sex.

### Carotid IMT in SSNHL and control group females of different ages

We further explored whether age influenced the association between IMT and SSNHL in females (Table 3). In women ≥50 years of age, the common cardiovascular factor values did not differ between the SSNHL and control groups. However, among females aged ≥50 years, average carotid IMT values were significantly higher in SSNHL patients (median, 0.72 mm; IQR, 0.62–1.08 mm) than those in the control group (median, 0.63 mm; IQR, 0.58–0.64 mm) (*P* = 0.016). Likewise, the percentage of female patients ≥50 years with high IMT (75%, 12 of 16) was significantly greater in the SSNHL group than the control group (25%, 2 of 8; OR 9.0 (95% CI [1.27–63.9])) (*P* = 0.032). In contrast, the median IMT value for female SSNHL patients aged <50 years and the proportion of female SSNHL patients aged <50 years with IMT ≥0.65 mm did not differ significantly from those in the control group. The percentage of male SSNHL patients and control subjects with IMT ≥0.65 mm did not differ significantly among those aged ≥50 years or <50 years.

**Table 3:** Characteristics of female SSNHL and control subjects stratified by 50 years of age. Female SSNHL patients ≥ 50 y/o had significant higher average IMT value and more percentage of high average IMT (≥ 0.65 mm) than those in the female control group ≥50 y/o. *P* values < 0.05 are considered statistically significant and are bolded.

	SSNHL	Control	*P* Value
Age, mean (SD), year			
≥50 y/o	62.2 (7.8)	56.9 (4.9)	0.095
<50 y/o	38.4 (11.3)	37.5 (10.6)	0.875
DM, *n* (%)			
≥50 y/o			
Yes	4 (25%)	0 (0%)	0.262
No	12 (75%)	8 (100%)	
<50 y/o			
Yes	0 (0%)	2 (25%)	0.467
No	8 (100%)	6 (75%)	
HTN, *n* (%)			
≥50 y/o			
Yes	6 (37.5%)	1 (12.5%)	0.352
No	10 (62.5%)	7 (87.5%)	
<50 y/o			
Yes	0 (0%)	2 (25%)	0.467
No	8 (100%)	6 (75%)	
Total cholesterol, mean (SD), mg/dl			
≥50 y/o	194.3 (37.9)	182.8 (42.6)	0.574
<50 y/o	202.8 (29.3)	205.8 (34.9)	0.873
LDL cholesterol, mean (SD), mg/dl			
≥50 y/o	116.1 (37.9)	96.2 (30.7)	0.304
<50 y/o	119.4 (36.3)	99.8 (28.7)	0.331
HDL cholesterol, median (IQR), mg/dl			
≥50 y/o	57 (53–68)	68 (49.5–82)	0.382
<50 y/o	58.5 (54.5–72)	70 (47–118.5)	0.769
Average IMT, median (IQR), mm			
≥50 y/o	0.72 (0.62–1.08)	0.63 (0.58–0.64)	**0.016**
<50 y/o	0.54 (0.47–0.59)	0.55 (0.48–0.56)	**0.752**
Average IMT, *n* (%)			
≥50 y/o			
High (≥0.65 mm)	12 (75%)	2 (25%)	**0.032**
Low (<0.65 mm)	4 (25%)	6 (75%)	
<50 y/o			
High (≥0.65 mm)	7 (87.5%)	8 (100%)	1.000
Low (<0.65 mm)	1 (12.5%)	0 (0%)	

**Note:**

SSNHL, sudden sensorineural hearing loss; DM, diabetes mellitus; HTN, hypertension; LDL, low-density lipoprotein; HDL, high-density lipoprotein; IMT, intima-media thickness; y/o, years old.

### Disease severity and treatment outcomes between SSNHL patients with low and high IMT

In terms of disease severity, the PTA and WRS of affected ears and associated vertigo symptoms did not differ significantly between the low and high IMT groups (Table 4). The treatment outcomes, evaluated by hearing gain, WRS change and Siegel’s criteria, did not differ significantly between the two groups (Table 4). Disease severity and treatment outcomes also did not differ between the low and high IMT groups when we focused on either female or male subjects.

**Table 4:** Comparison of disease severity and treatment outcomes between SSNHL patients with low and high IMT.

	Low IMT (<0.65 mm) (*n* = 22)	High IMT (≥0.65 mm) (*n* = 25)	*P* Value
PTA in affected ear, mean (SD), dB	72.8 (22.8)	72.1 (24.0)	0.915
WRS in affected ear, median (IQR), %	16 (0–93)	64 (0–92)	0.321
Vertigo, *n* (%)			
Yes	12 (54.5%)	8 (32%)	0.119
No	10 (45.5%)	17 (68%)	
Hearing gain, mean (SD), dB	23.3 (22.6)	24.2 (20.4)	0.892
WRS change, median (IQR), %	12 (0–74)	8 (0–60.5)	0.652
Siegel’s criteria (*n*, %)			
Complete recovery	5 (22.6%)	6 (24%)	0.696
Partial recovery	4 (18.2%)	7 (28%)	
Slight recovery	4 (18.2%)	2 (8%)	
No improvement	9 (41.0%)	10 (40%)	

**Note:**

IMT, intima-media thickness; PTA, pure tone average; dB, decibel; SD, standard deviation; WRS, word recognition score.

## Discussion

Diagnosed patients often ask about the cause of SSNHL. Unfortunately, there is a lack of tools that can detect auditory pathway injury, causing most SSNHL cases to be only suspected after taking clinical histories and examing risk factors. Gluco-metabolic, lipidic and coagulative data can identify vascular risk factors for SSNHL (Fasano et al., 2017), but not all laboratory tests are cost-effective (Heman-Ackah, Jabbour & Huang, 2010; Wilson et al., 2010). Here, we show that the higher carotid IMT is associated with an increased risk of SSNHL.

Carotid Doppler ultrasonography is commonly used to evaluate carotid artery atherosclerosis, an independent risk factor for stroke and myocardial infarction (Bots et al., 1997). In recent years, SSNHL has been associated with stroke (Kim, Hong & Kim, 2018a; Kim et al., 2018b; Lin, Chao & Lee, 2008) and myocardial infarction (Keller et al., 2013). These associations and the high frequency of cardiovascular risk factors in SSNHL patients (Aimoni et al., 2010) suggest that SSNHL is closely linked to cardiovascular disorders. Since the carotid system is the most important vascular supply for cerebral perfusion, it is reasonable to hypothesize that carotid atherosclerosis is a risk factor for SSNHL. Two prior studies found an increase in carotid IMT in SSNHL patients (Mutlu et al., 2018; Rajati et al., 2016). Likewise, we found a higher percentage of high-IMT (≥0.65 mm) subjects in the SSNHL group. In a previous study in Taiwan, the risk of atherosclerosis increased in subjects with carotid IMT >0.5 mm (Sun et al., 2002). Our work reveals early atherosclerotic changes in the carotid arteries of SSNHL patients.

Both sex and age influence the development of atherosclerosis and cardiovascular diseases (Spence & Pilote, 2015). We found that significantly more females with SSNHL had carotid IMT ≥0.65 mm compared to control group females, although the difference was not significant between SSNHL and control groups in the male subjects. This suggests that greater carotid IMT is a risk factor for female SSNHL patients. Our finding is in accordance with the results of a previous study that used mostly male subjects and found no significant difference in the carotid IMT of SSNHL and control groups (Ciccone et al., 2012). Recent studies have emphasized the influence of sex differences on cardiovascular risk factors (Appelman et al., 2015). Females have smaller carotid arteries than males (Krejza et al., 2006; Schulz & Rothwell, 2001). Therefore, early atherosclerotic changes may influence the vascular systems of end-organs, such as the inner ear, in females more than in males.

Another possible explanation for the greater carotid IMT in female SSNHL patients is the loss of estrogen during and after menopause. We found that the proportion of females aged ≥50 years with high carotid IMT was significantly greater in SSNHL patients than in control subjects. This was not the case for females aged <50 years. In Taiwan, the mean menopausal age is about 50 years (Chang, Chow & Hu, 1995). Therefore, early atherosclerosis during menopause may increase the risk of SSNHL. Estrogen has been found to have protective vascular effects, promoting vasodilation and exerting an antioxidative action that protects against vascular injury (Mendelsohn & Karas, 1999). Oxidative stress had been suggested as a risk factor for endothelial dysfunction and SSNHL (Capaccio et al., 2012; Ciccone et al., 2012; Quaranta, De Ceglie & D’Elia, 2016). Menopausal females with high IMT may be more predisposed to endothelial dysfunction and SSNHL than low-IMT subjects. Because post-menopausal women experience more microvascular dysfunction, endothelial dysfunction contributing to ischemic heart disease affects post-menopausal women more than premenopausal women (Kane & Howlett, 2018). Further studies with more subjects are required to clarify whether sex and age differences influence the effects of vascular factors and early atherosclerosis on SSNHL development.

To the best of our knowledge, this is the first study to explore the influence of carotid IMT on SSNHL treatment outcomes. We found no significant difference in treatment outcomes between the high- and low-IMT groups, evaluated either by hearing gain or using Siegel’s criteria. This was in agreement with the findings of previous studies which found that cardiovascular risk factors and metabolic syndromes did not affect SSNHL prognosis (Chien et al., 2015; Ciorba et al., 2015). It is probable that audiological factors, including the extent of initial hearing loss, the time elapsed between hearing loss onset and treatment, and associated vertigo, affect treatment outcomes (Chien et al., 2015; Ciorba et al., 2015) more profoundly than carotid IMT. Although increased carotid IMT does not influence SSNHL prognosis, it is unclear whether early atherosclerosis increases the risk of stroke in SSNHL patients. The associations between SSNHL and stroke have been studied (Kim, Hong & Kim, 2018a; Lin, Chao & Lee, 2008). Future studies should explore whether SSNHL patients (particularly postmenopausal females) with greater carotid IMT have a higher risk of stroke.

In 2014, the American Heart Association published guidelines for stroke prevention in females (Bushnell et al., 2014) since they exhibit more stroke risk factors than males, including obesity/metabolic syndrome, atrial fibrillation and migraines with auras. Our work emphasizes the importance of sex differences when considering SSNHL risk factors, including increased carotid IMT. Further prospective and populational studies may confirm whether postmenopausal women with increased carotid IMT have higher risks of SSNHL and stroke.

Our work has several limitations. First, this study is cross-sectional, not populational, so we could not estimate the associations between high carotid IMT and SSNHL in the total population. Second, we found a significant difference in high IMT frequency between SSNHL and control subjects when analyzing all, female, or older female subjects, but the relatively small number of females aged ≥50 years decreased our statistical power. Third, all control subjects underwent health examinations which may have detected higher proportions of cardiovascular risk factors than in the general population. For example, the prevalence of DM in our control group (12.1%) was higher than the prevalence in the general population (around 6%), as revealed by Taiwan’s National Health Insurance database (Jiang et al., 2012). Future large-scale, population-based, prospective cohort studies on SSNHL incidence in subjects with high carotid IMT could verify whether early atherosclerosis increases the risk of SSNHL.

## Conclusions

We found that carotid IMT was significantly associated with the risk of SSNHL, particularly in females aged ≥50 years, but not with treatment outcomes. Our findings provide insights for future studies on other large-scale prospective registries to assess the role of carotid IMT in SSNHL.

## Supplemental Information

10.7717/peerj.9276/supp-1 Supplemental Information 1 Raw data.

10.7717/peerj.9276/supp-2 Supplemental Information 2 Codebook.

## Additional Information and Declarations

### Data Availability

The following information was supplied regarding data availability: The raw data is available in the Supplementary Files.
